# Systemic and stratum corneum biomarkers of severity in infant atopic dermatitis include markers of innate and T helper cell‐related immunity and angiogenesis

**DOI:** 10.1111/bjd.17088

**Published:** 2018-10-04

**Authors:** M.A. McAleer, I. Jakasa, G. Hurault, P. Sarvari, W.H.I. McLean, R.J. Tanaka, S. Kezic, A.D. Irvine

**Affiliations:** ^1^ National Children's Research Centre Our Lady's Children's Hospital Crumlin Dublin 12 Ireland; ^2^ Paediatric Dermatology Our Lady's Children's Hospital Crumlin Dublin 12 Ireland; ^3^ Laboratory for Analytical Chemistry Department of Chemistry and Biochemistry Faculty of Food Technology and Biotechnology University of Zagreb Zagreb Croatia; ^4^ Department of Bioengineering Imperial College London London U.K.; ^5^ Dermatology and Genetic Medicine University of Dundee Dundee U.K.; ^6^ Amsterdam UMC University of Amsterdam Coronel Institute of Occupational Health Amsterdam Public Health research institute Meibergdreef 9 Amsterdam the Netherlands; ^7^ Clinical Medicine Trinity College Dublin Dublin Ireland

## Abstract

**Background:**

Biomarkers of atopic dermatitis (AD) are largely lacking, especially in infant AD. Those that have been examined to date have focused mostly on serum cytokines, with few on noninvasive biomarkers in the skin.

**Objectives:**

We aimed to explore biomarkers obtainable from noninvasive sampling of infant skin. We compared these with plasma biomarkers and structural and functional measures of the skin barrier.

**Methods:**

We recruited 100 infants at first presentation with AD, who were treatment naive to topical or systemic anti‐inflammatory therapies, and 20 healthy children. We sampled clinically unaffected skin by tape stripping the stratum corneum (SC). Multiple cytokines and chemokines and natural moisturizing factor were measured in the SC and plasma. We recorded disease severity and skin barrier function.

**Results:**

Nineteen SC and 12 plasma biomarkers showed significant differences between healthy and AD skin. Some biomarkers were common to both the SC and plasma, and others were compartment specific. Identified biomarkers of AD severity included T helper 2‐skewed markers [interleukin (IL)‐13, CCL17, CCL22, IL‐5]; markers of innate activation (IL‐18, IL‐1α, IL1β, CXCL8) and angiogenesis (Flt‐1, vascular endothelial growth factor); and others (soluble intercellular adhesion molecule‐1, soluble vascular cell adhesion molecule‐1, IL‐16, IL‐17A).

**Conclusions:**

We identified clinically relevant biomarkers of AD, including novel markers, easily sampled and typed in infants. These markers may provide objective assessment of disease severity and suggest new therapeutic targets, or response measurement targets for AD. Future studies will be required to determine whether these biomarkers, seen in very early AD, can predict disease outcomes or comorbidities.

## Abstract 2

**What's already known about this topic?**

- Atopic dermatitis is a clinically heterogeneous condition with multiple clinical manifestations and a complex pathogenesis.
- Systemic biomarkers of severity have been identified in adults, but are less well defined in children.
- Biomarkers from the skin compartment have been based on biopsies to date.

**What does this study add?**

- Noninvasive sampling can detect clinically relevant biomarkers in AD skin.
- These biomarkers may be useful for disease stratification, and provide insights into the pathogenesis of infant AD.
- Innate immune activation is important in the epidermis in infantile AD.

**What is the translational message?**

- Noninvasive biomarkers can yield significant insights into infantile AD.
- They identify innate activation, the T helper 2 pathway and angiogenesis as important pathways in this condition.

Respond to this article

**Linked Comment:** Hijnen. *Br J Dermatol* 2019; **180**:455–456.

Plain language summary available online

Atopic dermatitis (AD) is the most common inflammatory skin disease, and the most common inflammatory disease of childhood.^1^, ^2^ It affects up to 20% of children, and up to 10% of adults have a lifetime incidence of AD.^3^ The disease typically starts in infancy; in total 45% of all cases of AD begin within the first 6 months of life, 60% begin during the first year and 85% begin before 5 years of age.^3^ The importance of both a skin barrier defect and immune dysregulation in AD has been clearly demonstrated.^4^ Alterations and deficiencies in stratum corneum (SC) proteins, particularly filaggrin, and lipids have been implicated in the skin barrier deficiency.^4^, ^5^ Several other pathogenic factors such as dysbiosis and epigenetic modulation of the disease have been identified, adding further layers of complexity.^6^ AD has a wide spectrum of clinical presentations, natural history, severity, therapeutic response and associated diseases.^6^ This diversity of clinical phenotype likely reflects the complexity of the disease pathomechanisms.

There is generalized skin dysfunction in AD, with abnormalities in both affected and unaffected skin. Clinically unaffected skin has subclinical inflammation with skin barrier dysfunction, a proinflammatory cytokine milieu and lymphocytic infiltration.^7^ A chronic mild inflammation has been demonstrated in AD skin even in between flares.^8^ The role of type 2 immune activation in AD pathogenesis is well established; the T helper cell (Th)2 axis seems to be pathogenic across all subtypes of AD.^9^, ^10^ However, increasing complexity in the immunopathogenesis of AD has become apparent, with activation of the Th22, Th17/interleukin (IL)‐23 and Th1 cytokine pathways reported.^11^ Acute AD lesions have elevated Th2 and Th22 responses.^12^, ^13^ In chronic AD lesions Th2 and Th22 responses are increased along with Th1 axis activation.^14^, ^15^ Th17 levels have been shown to be upregulated in patients with both acute and chronic AD.^14^, ^16^ IL‐22 and IL‐31 have also been identified as cytokines of interest, playing a role in epidermal hyperplasia and itch, respectively.^17^, ^18^, ^19^

Despite AD being predominantly a disease of infancy and childhood, there are few studies investigating the paediatric immune profile. Paediatric and adult AD have clinical differences, including the distribution of lesions^20^ and microbiome abnormalities,^21^ differences that may point to pathomechanistic diversity. Paediatric studies have demonstrated a correlation between serum biomarkers and disease activity. Biomarkers include IL‐31, CCL17, CCL22, CCL27, eosinophils and IgE.^22^, ^23^, ^24^, ^25^ mRNA expression on DNA extracted from skin biopsies has been studied in paediatric AD.^26^

Th2 expansion has been shown in peripheral blood in paediatric AD, without other polar T‐cell subsets in the blood; in contrast, adult profiles showed Th2 and Th22 polarization.^27^ In infant skin there was a Th2 response but also innate and IL‐17‐related inflammation.^28^ Nonlesional, clinically unaffected skin in both adults and children has increased expression levels of cytokines with as high, or even higher levels in children compared with adults.^28^, ^29^

There is an ongoing need for therapeutic stratification of new and established treatments. Biomarker profiles will be central to this aim.^6^ As an example of this work, Thijs *et al*. reported biological heterogenicity in AD when they investigated biomarkers in adult patients, and demonstrated four unique clusters of biomarkers.^30^ Infantile AD is an important phenotype to characterize as it marks the commencement of the disease. Here we profiled both the plasma and SC in infants with AD. We analysed these biomarkers alongside eczema severity assessments and transepidermal water loss (TEWL). We identified clinically relevant biomarkers of AD severity, including novel markers, easily sampled and typed in infants.

## Patients and methods

### Study population

One hundred infants with AD were recruited, from November 2012 to November 2014, in a dedicated AD clinic in Our Lady's Children's Hospital, Dublin. A single, experienced paediatric dermatologist (M.A.McA.) assessed, recruited and treated all patients. Patients had to be < 12 months of age with moderate or severe AD, as determined by a Scoring Atopic Dermatitis (SCORAD) score of ≥ 25, for ≥ 6 weeks’ duration. Furthermore, the patients had to be treatment naive, apart from the use of emollients and hydrocortisone 1% cream or ointment.

Twenty control patients were recruited when attending Our Lady's Children's Hospital, Dublin, for elective procedures under general anaesthetic. Patients were recruited if they did not have AD, any history suggestive of AD or any other inflammatory skin disease. All infants and children were examined to ensure an absence of inflammatory skin disease. All patients were asked to refrain from application of any topical agents for 24 h prior to assessment. The study was conducted in accordance with the Declaration of Helsinki and was approved by the research ethics committee of Our Lady's Children's Hospital, Dublin. Written informed consent was obtained from all patients’ parents.

### Clinical assessment

The patients met the Hanifin and Rajka criteria for the diagnosis of AD.^31^ The age of onset of AD was recorded. Severity was assessed using the SCORAD scale.^32^ All patients had moderate or severe AD defined by SCORAD ≥ 25. Objective SCORAD (oSCORAD) is derived from the SCORAD by not including subjective scores of parental assessment of sleep loss and itch.

### Stratum corneum transepidermal water loss measurement

TEWL measurements were done under standardized conditions (room temperature of 22–25 °C and humidity levels of 30–35%). Patients were acclimatized for a minimum of 10 min, with their volar forearm skin exposed. Measurements were taken from an area of clinically unaffected skin on the volar forearm using the Tewameter 300 (Courage + Khazaka electronic GmbH, Cologne, Germany).

### Sampling of the stratum corneum by tape stripping

The SC was sampled using the previously described method,^33^ using circular adhesive tape strips (3·8 cm^2^, D‐Squame; Monaderm, Monaco) and a D‐Squame pressure instrument D500 (CuDerm, Dallas, TX, U.S.A.). Eight consecutive tape strips were sampled, all from the same site, in nonlesional skin, 2 cm away from viable eczematous areas, and immediately stored at −80 °C.

### Blood sampling

Plasma was separated by centrifugation, pipetted into cryotubes and stored frozen at −80 °C until analysis.

### Determination of filaggrin breakdown products in the stratum corneum

Natural moisturizing factor (NMF) component analysis (histidine, pyrrolidone carboxylic acid, *trans*‐ and *cis*‐urocanic acid) and proteins was performed on the fourth consecutive strip according to the method previously described.^33^

### *FLG* genotyping

All patients were screened for the nine most common filaggrin mutations found in the Irish population (R501X, Y2092X, 2282del4, R2447X, S3247X, R3419X, 3702X, S1040X and G1139X), as previously described.^34^

### Cytokine analysis in tape strips and plasma samples

Cytokine concentrations in the SC and plasma were measured using MESO QuickPlex SQ 120 (MSD, Rockville, MA, U.S.A.) according to the manufacturer's instructions, apart from the samples being undiluted, and in the case of SC the sample incubation time was extended to 16 h.

Cytokines were measured on preconfigured multiplex panels, as follows. Proinflammatory panel: IL‐1β, IL‐2 and IL‐13 in the SC and additionally interferon‐γ, IL‐4, IL‐6, IL‐10, IL‐12p70 and tumour necrosis factor (TNF)‐α in plasma. Chemokine panel: CCL2, CCL3, CCL4, CCL13, CCL17, CCL22, CXCL8 and CXCL10 in the SC, and CCL5 and CCL11 in plasma. Cytokine panel: granulocyte–macrophage colony‐stimulating factor (GM‐CSF), IL‐1α, IL‐5, IL‐7, IL‐12p40, IL‐15, IL‐16 and IL‐17A in the SC, and TNF‐β, IL‐17A and vascular endothelial growth factor (VEGF) in plasma. Vascular panel: C‐reactive protein, serum amyloid A (SAA), soluble intercellular adhesion molecule (sICAM)‐1 and soluble vascular cell adhesion molecule (sVCAM)‐1 in both SC and plasma. Angiogenesis panel: Flt‐1, Tie‐2, VEGF‐A and VEGF‐C in the SC, and basic fibroblast growth factor, placental growth factor and VEGF‐D in plasma, as well as some singleplex assays (IL‐18).

For statistical analysis, cytokine concentrations below the detection limit (but above the bottom of the curve) or above the detection limit were taken unchanged, and cytokine concentrations that were below the fit curve range (signal below the bottom of the bottom‐of‐the‐curve fit, no concentration given) were assigned half the value of the lowest sample concentration below the detection limit to maintain the ranking order. The limits of detection are given in Table S1 (see Supporting Information).

### Extraction of cytokines from the stratum corneum

The fifth consecutive tape strip was used to measure cytokine levels in the SC. To determine the amount of soluble protein and cytokines, 0·6 mL of phosphate‐buffered saline (Merck, Darmstadt, Germany) with 0·005% Tween‐20 (Sigma‐Aldrich, Zwijndrecht, the Netherlands) was added to each vial, and the vials were left on ice for 30 min. Extraction was performed with an ultrasound sonifier equipped with a probe (Salm & Kipp, Breukelen, the Netherlands) for 15 min in ice water. The extract was centrifuged (2 min at 15 000 *g*), and supernatant aliquots of 60 μL were frozen at −80 °C until further analysis. The amount of cytokine in the SC was normalized by the protein content, which was determined using the Pierce Micro BCA Protein Assay Kit (Thermo Fischer Scientific, Rockford, IL, U.S.A.), with the bovine serum albumin supplied as standard.

### Statistical analysis

All calculations were performed using Prism 7 software (GraphPad, La Jolla, CA, U.S.A.) and R 3.4.0 (R Foundation for Statistical Computing, Vienna, Austria). The distribution of data was tested by the Shapiro–Wilk normality test; *P*‐values were corrected for multiple testing using a Benjamini–Hochberg procedure.^35^ The applied statistical test is indicated within the figure or the figure legends.

## Results

### Study patients

We recruited 100 infants with moderate or severe AD and 20 healthy control infants. From this cohort, we analysed plasma of 47 patient samples and 20 control samples. SC samples were analysed for NMF in 74 patient samples and 18 controls, and for cytokines in 66 patient samples and 13 controls. Demographic details of the patients and controls analysed in this study are outlined in Table 1. All raw study data are listed in Tables S2 and S3 (see Supporting Information).

**Table 1:** Demographic and laboratory details of the study participants

	Patients with AD		Controls	
	Plasma	SC	Plasma	SC
Total	47	66	20	13
Sex				
Male	34	49	14	9
Female	13	17	6	4
Age (months)				
Average	6·7	7	6·5	6·7
Range	0–11	0–11	0–12	0–12
Age at AD onset (weeks)				
Average	11·8	11	–	–
Range	2–44	2–44	–	–
SCORAD				
Average	48	45·2	–	–
Range	25–91·3	25–85	–	–
TEWL (g m^−2^ h^−1^)				
Average	26	24·1	11	12
Range	8·5–53·9	8·3–53·9	4–15·6	8·1–15·6
*FLG* status				
Wild‐type	26	30	18	11
Heterozygous	18	27	2	2
Homozygous	3	5	0	0
Unknown	1	4	0	0

AD, atopic dermatitis; SC, stratum corneum; SCORAD, Scoring Atopic Dermatitis; TEWL, transepidermal water loss.

### Measures of barrier function: unaffected atopic dermatitis skin vs. healthy control skin

**Figure 1:**
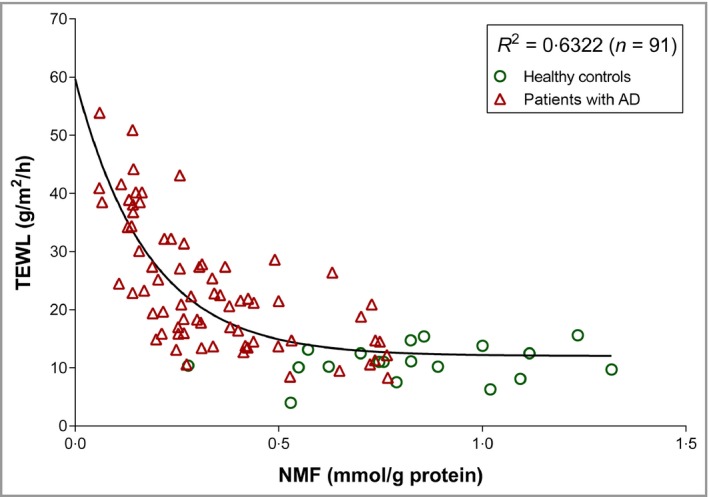
Relationship (exponential decay) between transepidermal water loss (TEWL) and natural moisturizing factor (NMF) in children with atopic dermatitis (AD) and healthy controls.

Patients with AD had significantly lower NMF levels and higher TEWL readings than healthy controls (Fig. S1; see Supporting Information). TEWL values in patients with AD and healthy controls decreased exponentially with NMF (Fig. 1). TEWL was associated with AD severity scores (Fig. S3; see Supporting Information).

### Stratum corneum and systemic biomarkers: atopic dermatitis vs. healthy controls

**Figure 2:**
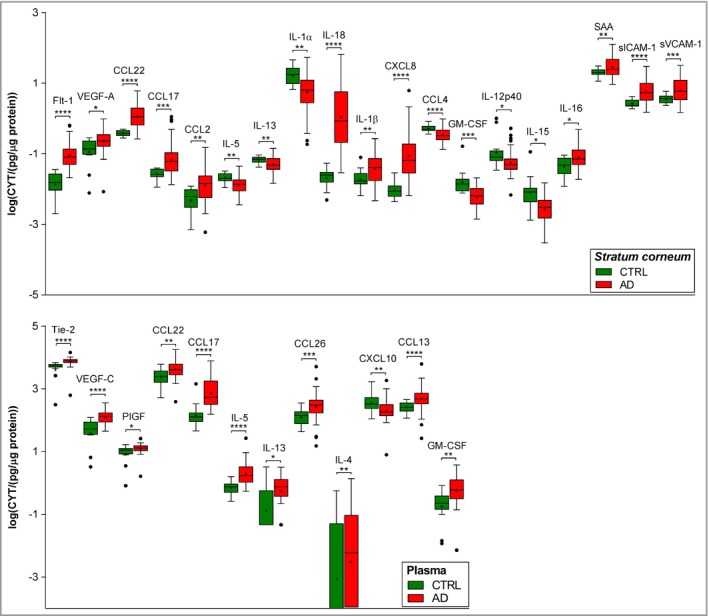
Levels of cytokines and chemokines in the stratum corneum of children with AD and healthy controls (CTRL). Stratum corneum: *n* = 66 (AD) and *n* = 13 (healthy); plasma: *n* = 47 (AD) and *n* = 20 (healthy). The values are log transformed, shown as boxplots with Tukey‐style whiskers. Differences in cytokine and chemokine levels between healthy children and children with AD were determined by two‐tailed Welch's *t*‐test or two‐tailed Mann–Whitney test (raw data in Table S2; see Supporting Information). Benjamini–Hochberg corrected *P‐*values: ****P < 0·0001, ***P < 0·001, **P < 0·01, *P < 0·05. GM‐CSF, granulocyte–macrophage colony‐stimulating factor; IL, interleukin; PIGF, placental growth factor; SAA, serum amyloid A; sICAM, soluble intercellular adhesion molecule; sVCAM, soluble vascular cell adhesion molecule; VEGF, vascular endothelial growth factor.

Nineteen of 27 and 12 of 39 measured biomarkers, in SC and plasma, respectively, showed significantly different levels in infants with AD compared with healthy controls (Fig. 2). While some biomarkers showed directional change (i.e. parallel increase or decrease in patients with AD vs. healthy controls), some of them showed discordance between SC and plasma, or were significantly different only in SC or plasma. For example, CCL17 and CCL22 were elevated in AD in both SC and plasma. However, several inflammatory biomarkers were significantly different from healthy controls only in the SC; these included IL‐1α, IL‐1β, IL‐15, IL‐16, IL‐18, IL‐12p40, SAA; chemokines CXCL8, CCL2 and CCL4; cell adhesion biomarkers sVCAM‐1 and sICAM‐1 and vascular factors VEGF‐A and Flt‐1. In contrast, VEGF‐C, Tie‐2 and CCL13 were significantly elevated in plasma and undetectable or unchanged in the SC (Fig. 2).

**Figure 3:**
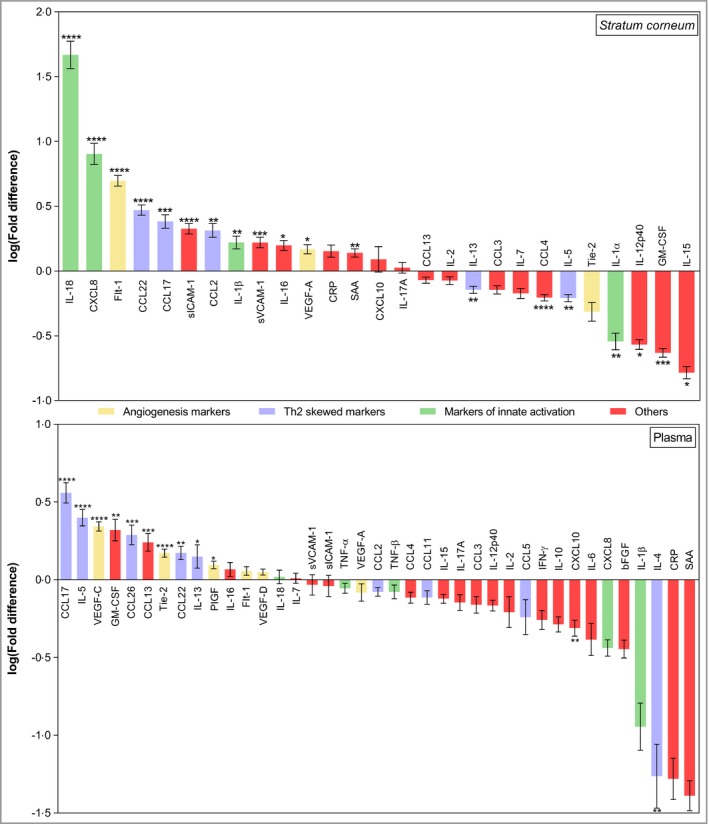
Fold differences between cytokine and chemokine levels in the stratum corneum and in plasma of children with atopic dermatitis (AD), and corresponding cytokine and chemokine levels in healthy children. The results are expressed as the mean ± SEM. Stratum corneum: *n* = 66 (AD) and *n* = 13 (healthy); plasma: *n* = 47 (AD) and *n* = 20 (healthy). The fold difference was calculated by dividing the individual cytokine or chemokine level in the SC and plasma of each child with AD by the corresponding mean cytokine or chemokine level in healthy children. Differences in cytokine and chemokine levels between healthy children and children with AD were determined by two‐tailed Welch's *t*‐test or two‐tailed Mann–Whitney test (raw data in Table S2; see Supporting Information). Benjamini–Hochberg corrected *P*‐values: ****P < 0·0001, ***P < 0·001, **P < 0·01, *P < 0·05. bFGF, basic fibroblast growth factor; CRP, C‐reactive protein; GM‐CSF, granulocyte–macrophage colony‐stimulating factor; IFN, interferon; IL, interleukin; PIGF, placental growth factor; SAA, serum amyloid A; sICAM, soluble intercellular adhesion molecule; sVCAM, soluble vascular cell adhesion molecule; Th, T helper cell; TNF, tumour necrosis factor; VEGF, vascular endothelial growth factor.

The biomarker profiles in plasma and SC not only differed qualitatively but also showed distinct magnitudes of difference. Figure 3 demonstrates the fold changes in SC and plasma biomarkers in infants with AD compared with healthy controls. In the skin compartment the most significantly elevated biomarkers in patients with AD were IL‐18, CXCL8, Flt‐1, CCL22, CCL17 and sICAM‐1. The most significant decreases were observed in IL‐15, GM‐CSF, CCL4, IL‐12p40, IL‐1α, IL‐13 and IL‐5. In plasma, the biomarkers CCL17, IL‐5, VEGF‐C, GM‐CSF and CCL26 had the most significant fold increases in plasma of patients with AD compared with healthy controls, while IL‐4 and CXCL10 had the most significant decreases. There was no significant differences in SC or plasma biomarkers following stratification for *FLG* mutations (Fig. S3; see Supporting Information) or by the clinical presence or absence of bacterial superinfection of AD (data not shown).

### Stratum corneum and systemic biomarkers: correlation with skin barrier function and disease severity

**Figure 4:**
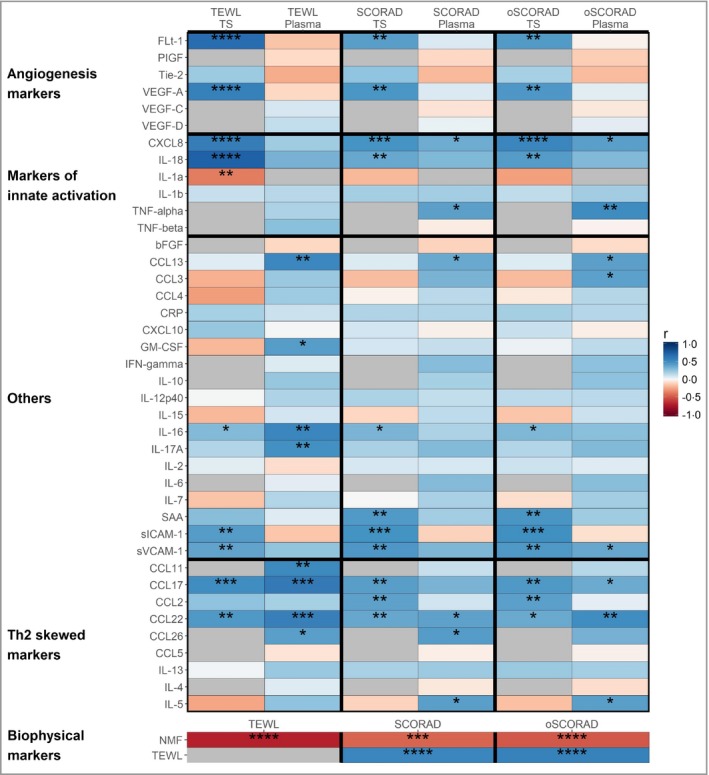
Spearman correlation coefficients between stratum corneum (tape stripping, TS) and plasma biomarkers and atopic dermatitis severity [Scoring Atopic Dermatitis (SCORAD) and objective SCORAD] and transepidermal water loss (TEWL). Benjamini–Hochberg corrected *P*‐values: ****P < 0·0001, ***P < 0·001, **P < 0·01, *P < 0·05. bFGF, basic fibroblast growth factor; CRP, C‐reactive protein; GM‐CSF, granulocyte–macrophage colony‐stimulating factor; IFN, interferon; IL, interleukin; NMF, natural moisturizing factor; PIGF, placental growth factor; SAA, serum amyloid A; sICAM, soluble intercellular adhesion molecule; sVCAM, soluble vascular cell adhesion molecule; Th, T helper cell; TNF, tumour necrosis factor; VEGF, vascular endothelial growth factor.

Correlation analysis between biomarkers and the biophysical measures of barrier function (TEWL) and AD severity (SCORAD and oSCORAD) are shown in Figure 4. Consistently with the differences found between healthy controls and patients with AD (Figs 2, 3), SC inflammatory biomarkers IL‐18, CXCL8, VEGF‐A and Flt‐1 showed the strongest associations with barrier function and AD severity, followed by CCL17, CCL22, sICAM‐1 and sVCAM‐1. In general, similar strength of biomarker association was obtained with SCORAD and oSCORAD. In plasma, biomarkers involved in the Th2 response, CCL22, CCL17 and CCL11, had the strongest association with barrier function. Other plasma inflammatory biomarkers associated with barrier function were CCL13, IL‐16 and IL‐17A (Fig. 4). Among plasma biomarkers, CCL22 and TNF‐α showed the strongest associations with disease severity (oSCORAD). The skin barrier parameters TEWL and NMF showed significant correlation with disease severity (Fig. 4 and Fig. S2; see Supporting Information).

## Discussion

This study is novel in several aspects. We studied a large sample size of treatment‐naive infants at initial presentation of moderate‐to‐severe AD. Our noninvasive technique has clear advantages when studying skin disease in any group, but particularly in infants and children. A diverse range of significantly associated and clinicopathologically relevant biomarkers could be measured in the SC. The skin and systemic compartments had distinct biomarker profiles; the skin compartment in our study reflects cytokines dominant in the outer layers of the SC, whereas the plasma profile reflects both tissue and systemic responses. The SC sampling we employed is, compared with full‐thickness biopsy, selective for the epidermis and thus more representative of mature cytokine protein expression in this compartment than whole‐biopsy mRNA. The techniques we employed to measure biomarkers require further validation; however, in this exploratory study they have proved promising. The identified biomarkers could broadly be grouped into those critical in innate immune activation, Th2 immune activation and angiogenesis.

The role of innate immune activation in the pathogenesis of infantile AD is supported by our findings of elevated IL‐18 and IL‐1β in the SC of patients with AD compared with healthy controls. These findings are in keeping with recent reports in early‐onset AD, where changes in the innate immune system were already detectable at birth.^36^ IL‐18 levels were particularly striking in our cohort: they were observed in very high levels in the SC, were the most significantly increased biomarker compared with healthy controls, were strongly associated with barrier function, and also correlated with disease severity. IL‐18 is a member of the IL‐1 family of cytokines.^37^ Elevated expression of serum IL‐18 is seen in children and murine models.^38^, ^39^ It has been shown to be elevated in the SC of adults with AD^40^, ^41^ and in the serum of infants and children with AD, correlating with disease activity.^42^, ^43^ We also found CXCL8 (IL‐8) to be significantly increased in the SC and correlated with disease severity and barrier dysfunction, further supporting the role of innate activation. The pathomechanistic role of CXCL8 in AD has not been studied. SC levels of CXCL8 have been shown to be closely correlated with SC IL‐18; however, their relationship, if any, in AD is unknown.^44^

Many cytokines and chemokines involved in the Th2 response were elevated in our cohort. CCL17 and CCL22 were significantly elevated in both the skin and plasma, had notable fold changes compared with controls, and were significantly correlated with severity and barrier function. CCL17, produced by Th2 cells and keratinocytes, is a key chemokine involved in homing of CCR4‐expressing T cells to the skin.^45^ CCL17 has been detected in the inflamed skin of patients and in an animal model of AD.^46^, ^47^ Serum CCL17 is strongly correlated with disease severity in patients with AD, including infants, and is the most reliable objective AD biomarker identified to date.^48^, ^49^, ^50^, ^51^ CCL22 is similar to CCL17 and is a chemoattractant for CCR4‐expressing skin‐homing T cells.^46^

Meta‐analysis of available studies reported a strong correlation coefficient between CCL22 and disease severity in AD.^51^ IL‐13 was elevated in the plasma of infants with AD. The role of IL‐13 has been extensively studied in AD, in both animal models and humans, and its role in AD pathogenesis is well established. The other prototypical type 2 cytokine is IL‐4, and its aberrant production has long been associated with atopic disorders. We were unable to detect IL‐4 in high enough levels to measure it accurately. IL‐13 levels have often been shown to be elevated compared with IL‐4 levels in inflamed tissues.^52^ Murine work suggests that this is because primary sources of IL‐4 include basophils and conventional Th2 cells,^53^ in contrast to tissue Th2 cells and group 2 innate lymphoid cells (ILC2s), which are the major producers of IL‐13.^54^, ^55^ The fact that ILC2s produce significant quantities of IL‐13, but not IL‐4, may explain the quantities and timing of expression of these cytokines.^52^

We demonstrated significantly decreased IL‐5 levels in the skin compartment of patients with AD compared with healthy controls, in contrast to significantly increased levels in the plasma that positively correlated with disease severity scores. Th2 cells and mast cells are the major IL‐5‐producing cells.^56^, ^57^ ILC2s also produce Th2 cytokines, including IL‐5. ILC2s are involved in innate immune responses in allergy and infection.^58^ Unlike in atopic lung disease, IL‐5 has not been studied extensively in AD. Gürkan *et al*. reported significantly increased serum IL‐5 levels in infants with AD compared with controls, but did not demonstrate a correlation with disease severity.^59^ Eosinophils constitutively express IL‐31RA and release proinflammatory cytokines and chemokines, including IL‐1β, IL‐6, IL‐31, CXCL1, CXCL8, CCL2, CCL18 and CCL26, in response to IL‐31.^60^ In keeping with elevated IL‐5 levels and eosinophil activation, we found elevated levels of CCL2, IL‐1β and CXCL8 in the SC.

The third prominent biomarker profile in our cohort identifies angiogenesis and lymphangiogenesis as key processes. Biomarkers for these were significantly elevated in patients compared with controls and correlated with disease severity and barrier function. Angiogenesis is a hallmark of chronic inflammatory skin diseases and plays a role in AD, although this has not been extensively studied.^61^ Plasma and skin concentrations of VEGF‐A are increased in patients with AD compared with controls.^62^, ^63^ The function of lymphangiogenesis in AD and whether it represents a feasible therapeutic target is unclear.^61^ It is possible that the biological role of lymphangiogenesis in AD may not be pathogenic, but instead a response to inflammation, attempting to rectify the disease through transportation of allergens and inflammatory cells away from the inflamed skin.^64^, ^65^

Other biomarkers of note in our cohort were GM‐CSF and sICAM‐1. GM‐CSF levels were diminished in the SC and increased in the plasma of infants with AD in our cohort. Plasma levels negatively correlated with barrier function. GM‐CSF is synthesized and released by multiple cutaneous cells including keratinocytes.^66^

Esaki *et al*. investigated early‐onset paediatric AD biomarkers, using immunohistochemistry and real‐time polymerase chain reaction to study skin biopsies from 19 children.^28^ The children were within 6 months of disease onset, with a mean age of 1·3 years. The authors reported Th17, Th9, Th2 and Th22 activation in both lesional and nonlesional skin and demonstrated significantly higher induction of Th17‐related cytokines (IL‐17A, IL‐19, CCL20, LL‐37 and peptidase inhibitor 3) in children with AD compared with adults with AD and control patients.^28^ We did not demonstrate any statistically significant difference in IL‐17A levels in the SC or plasma of patients with AD compared with healthy controls. However, we did find an association between IL‐17A levels and barrier function, in keeping with a recent murine study.^67^ It is notable that in the study of Esaki *et al*. the statistically significant difference in IL‐17A levels in paediatric skin was observed when lesional skin was compared with control children or skin of adults with AD. There was no significant difference observed between IL‐17A levels in control children and nonlesional skin in children with AD,^28^ and hence our IL‐17A findings concur with their results.

AD is a ‘multiaxis’ immune disease. It is becoming apparent that different subtypes of the disease are associated with variations in immune profiles. Single‐cytokine targeting may not be fully effective in this complex disorder, and multicytokine therapies or combination treatment may prove more effective. Therapeutic options in AD are poised to increase rapidly, along with drug costs. An objective and evidence‐based approach with respect to disease severity and therapeutic response will become even more relevant. Combinations of biomarkers with good predictive ability identified by multidimensional data and multivariate analysis may be useful tools in drug selection and justification in our patients with severe AD.

We have demonstrated an SC and plasma biomarker profile for infants with moderate‐to‐severe AD, a novel study in this age group. Innate immunity, Th2 responses and lymphangiogenesis appear to be important mechanisms in early disease. Further understanding of the predictive abilities of biomarker combinations may have clinical utility in objective severity measurements, disease prognostication and measuring therapeutic responses.

## Supporting information


**Table S1** Cytokine and chemokine limits of detection and number of cytokines with concentrations below the fit curve range in the stratum corneum and in plasma.
**Table S2** Cytokine and chemokine levels (log‐transformed values) and differences between their levels in the stratum corneum and plasma of healthy control children and children with atopic dermatitis.
**Table S3** Correlation (two‐tailed Spearman's test) between cytokines and chemokines (log‐transformed values) and (a) Scoring Atopic Dermatitis (SCORAD)/objective SCORAD and (b) transepidermal water loss in the stratum corneum and in plasma of children with atopic dermatitis.

**Fig S1.** Natural moisturizing factor levels in the stratum corneum, and transepidermal water loss in children with atopic dermatitis and healthy controls.

**Fig S2.** Linear regression for transepidermal water loss and atopic dermatitis severity [Scoring Atopic Dermatitis (SCORAD) and objective SCORAD] in children with atopic dermatitis.

**Fig S3.** Levels of cytokines and chemokines, natural moisturizing factor and transepidermal water loss in healthy controls and children with atopic dermatitis stratified for *FLG* mutations.
