# Supplementary material for: Systemic and stratum corneum biomarkers of severity in infant atopic dermatitis include markers of innate and T helper cell‐related immunity and angiogenesis
Source: Br J Dermatol. 2018 Oct 4;180(3):586–96. doi: 10.1111/bjd.17088 (PMC6446820; doi:10.1111/bjd.17088)
Supplement: Supplementary file 1 — Table S1 Cytokine and chemokine limits of detection and number of cytokines with concentrations below the fit curve range in the stratum corneum and in plasma. Table S2 Cytokine and chemokine levels (log‐transformed values) and differences between their levels in the stratum corneum and plasma of healthy control children and children with atopic dermatitis. Table S3 Correlation (two‐tailed Spearman's test) between cytokines and chemokines (log‐transformed values) and (a) Scoring Atopic Dermatitis (SCORAD)/objective SCORAD and (b) transepidermal water loss in the stratum corneum and in plasma of children with atopic dermatitis. [file BJD-180-586-s001.docx]

**Table S1.** Cytokine and chemokine limits of detection (LoD) and number of cytokines with concentrations below the fit curve range in the stratum corneum (SC) and in plasma.

| **CYT** | **LoD** | |  | **Number of cytokines with concentrations below fit curve range** | | | |
| --- | --- | --- | --- | --- | --- | --- | --- |
|  | **SC** | **Plasma** |  | **SC** | | **Plasma** | |
|  | **pg/mL** | |  | **Ctrl group** | **AD group** | **Ctrl group** | **AD group** |
| Flt-1 | 1.45 | 0.24 |  | 1 | 0 | 0 | 0 |
| Tie-2 | 4.51 | 14.7 |  | 0 | 10 | 0 | 2 |
| VEGF-A | 1.39 | 1.15 |  | 1 | 1 | 0 | 0 |
| VEGF-C | 13.1 | 19.4 |  | 10 | 60 | 0 | 3 |
| PIGF | nd | 0.1 |  | Nd | nd | 0 | 0 |
| VEGF-D | nd | 3.8 |  | Nd | nd | 0 | 0 |
| CCL2 (MCP-1) | 0.109 | 5.34 |  | 0 | 0 | 0 | 0 |
| CCL22 (MDC) | 3.0 | 3.29 |  | 0 | 0 | 0 | 0 |
| CCL17 (TARC) | 0.106 | 0.19 |  | 0 | 0 | 0 | 0 |
| IL-5 | 0.114 | 0.11 |  | 0 | 0 | 0 | 0 |
| IL-13 | 0.619 | 0.31 |  | 0 | 0 | 7 | 12 |
| CCL26 (Eotaxin-3) | nd | 0.77 |  | nd | nd | 0 | 0 |
| CCL11 (Eotaxin) | nd | 2.14 |  | nd | nd | 0 | 0 |
| IL-4 | nd | 0.08 |  | nd | nd | 13 | 21 |
| CCL5 (RANTES) | nd | 0.62 |  | nd | nd | 2 | 2 |
| IL-1α | 0.202 | 0.6 |  | 0 | 0 | 9 | 26 |
| IL-18 | 1.22 | 0.74 |  | 0 | 0 | 0 | 0 |
| IL-1β | 0.0894 | 0.04 |  | 0 | 0 | 19 | 7 |
| CXCL8 (IL-8) | 0.0457 | 0.06 |  | 0 | 0 | 0 | 0 |
| TNF-β | nd | 0.07 |  | nd | nd | 0 | 0 |
| TNF-α | nd | 0.09 |  | nd | nd | 0 | 0 |
| CXCL10 (IP-10) | 0.206 | 0.26 |  | 5 | 7 | 0 | 0 |
| CCL13 (MCP-4) | 4.23 | 2.38 |  | 0 | 0 | 0 | 0 |
| CCL3 (MIP-1α) | 4.35 | 5.34 |  | 0 | 3 | 4 | 0 |
| CCL4 (MIP-1β) | 5.6 | 2.95 |  | 0 | 1 | 0 | 0 |
| GM-CSF | 0.131 | 0.1 |  | 0 | 0 | 1 | 0 |
| IL-7 | 0.19 | 0.16 |  | 0 | 0 | 0 | 0 |
| IL-12p40 | 1.08 | 0.53 |  | 0 | 0 | 0 | 0 |
| IL-15 | 0.129 | 0.11 |  | 0 | 1 | 0 | 0 |
| IL-16 | 0.747 | 0.53 |  | 0 | 0 | 0 | 0 |
| IL-17A | 0.633 | 0.69 |  | 0 | 1 | 0 | 0 |
| IL-2 | 0.0894 | 0.06 |  | 0 | 0 | 6 | 4 |
| CRP | 73 | 3.74 |  | 0 | 0 | 1 | 0 |
| SAA | 555 | 43.2 |  | 0 | 0 | 1 | 0 |
| sICAM-1 | 76 | 2.66 |  | 0 | 0 | 0 | 0 |
| sVCAM-1 | 107 | 5.55 |  | 0 | 0 | 0 | 0 |
| bFGF | nd | 0.07 |  | nd | nd | 0 | 0 |
| IFN-γ | nd | 0.69 |  | nd | nd | 0 | 0 |
| IL-6 | nd | 0.14 |  | nd | nd | 1 | 1 |
| IL-10 | nd | 0.09 |  | nd | nd | 0 | 0 |

nd → not determined in the *SC*

| Angiogenesis markers | Th2 skewed markers | Markers of innate activation | Others |
| --- | --- | --- | --- |

**Table S2** Cytokine and chemokine levels (log-transformed values) and differences between their levels in the *SC* and plasma of healthy control children

(CTRL) and children with AD (AD).

|  | ***SC*** | | |  | **Plasma** | | |
| --- | --- | --- | --- | --- | --- | --- | --- |
|  | **CTRL (*n* = 13)** | **AD (*n* = 66)** | **Ctrl vs. AD** |  | **CTRL (*n* = 20)** | **AD (*n* = 47)** | **Ctrl vs. AD** |
|  | *median*(range) | *median*(range) | (adjusted *p*-value) |  | *median*(range) | *median*(range) | (adjusted *p*-value) |
| Flt-1 | -1,812(-2,699 to -1,449) | -1,095(-1,684 to -0,195) | < 0.0001 ^(1)^ |  | 1,460(-0,387 to 1,648) | 1,530(0,524 to 1,810) | 0.1371 |
| Tie-2 | -1,234(-1,588 to -0,452) | -1,204(-2,721 to -0,539) | 0.9162 |  | 3,735(2,493 to 3,834) | 3,892(2,794 to 4,160) | < 0.0001 |
| VEGF-A | -0,864(-2,108 to-0,552) | -0,637(-2,071 to -0,010) | 0.0200 |  | 1,488(0,961 to 2,048) | 1,578(0,649 to 2,227) | 0.9332 ^(1)^ |
| VEGF-C |  |  | < DL |  | 1,725(0,513 to 2,092) | 2,135(1,650 to 2,554) | < 0.0001 |
| CCL2 (MCP-1) | -2,208(-3,155 to-1,917) | -1,845(-3,222 to -0,821) | 0.0055 ^(1)^ |  | 2,269(1,913 to 2,527) | 2,208(1,655 to 2,627) | 0.2641 ^(1)^ |
| CCL22 (MDC) | -0,425(-0,555 to-0,306) | 0,046(-0,577 to 0,785) | < 0.0001 ^(1)^ |  | 3,406(2,717 to 3,781) | 3,606(2,588 to 4,256) | 0.0071 |
| CCL17 (TARC) | -1,580(-1,947 to-1,409) | -1,221(-1,876 to 0,051) | 0.0007 |  | 2,103(1,661 to 3,159) | 2,750(2,189 to 3,894) | < 0.0001 |
| IL-5 | -1,699(-1,959 to-1,491) | -1,863(-2,444 to -1,350) | 0.0022 ^(1)^ |  | -0,124(-0,588 to 0,200) | 0,176(-0,263 to 1,428) | < 0.0001 |
| IL-13 | -1,154(-1,381 to-1,035) | -1,320(-1,839 to -0,847) | 0.0052 ^(1)^ |  | -1,331(-1,331 to 0,509) | -0,115(-1,331 to 0,500) | 0.0133 |
| IL-1α | 1,252(0,820 to1,662) | 0,805(-0,731 to 1,730) | 0.0022 |  |  |  | < DL |
| IL-18 | -1,606(-2,310 to-1,271) | -0,063(-1,536 to 1,817) | < 0.0001 ^(1)^ |  | 3,160(2,887 to 3,717) | 3,334(2,113 to 3,692) | 0.1906 |
| IL-1β | -1,767(-2,187-1,102) | -1,395(-2,328 to -0,571) | 0.0097 ^(1)^ |  | -1,080(-2,770 to 0,294) | -1,340(-2,770 to -0,164) | 0.3598 |
| CXCL8 (IL-8) | -2,060(-2,357 to-1,541) | -1,185(-2,187 to 0,794) | < 0.0001 |  | 1,036(0,550 to 2,662) | 1,113(0,367 to 1,983) | 0.6895 |
| CXCL10 (IP-10) | -2,721(-4,000 to-1,851) | -2,310(-4,000 to -0,418) | 0.0785 |  | 2,513(2,050 to 3,223) | 2,263(0,898 to 3,268) | 0.0097 |
| CCL13 (MCP-4) | -0,564(-0,798 to-0,377) | -0,601(-1,214 to -0,255) | 0.3598 ^(1)^ |  | 2,406(2,073 to 2,657) | 2,685(1,425 to 3,795) | 0.0005 |
| CCL3 (MIP-1α) | -0,761(-1,078 to-0,578) | -0,890(-1,710 to -0,369) | 0.1473 |  | 1,400(0,339 to 1,665) | 1,336(0,339 to 1,964) | 0.2779 |
| CCL4 (MIP-1β) | -0,310(-0,442 to-0,084) | -0,490(-0,881 to -0,014) | < 0.0001 ^(1)^ |  | 1,957(1,526 to 2,158) | 1,814(1,267 to 2,343) | 0.1507 ^(1)^ |
| GM-CSF | -1,857(-2,114 to-0,789) | -2,194(-2,854 to -1,686) | 0.0002 |  | -0,647(-1,928 to -0,082) | -0,217(-2,143 to 0,576) | 0.0010 |
| IL-7 | -1,928(-2,824 to-1,578) | -1,971(-3,046 to -1,654) | 0.4594 |  | 0,906 (0,617 to 1,189) | 0,952(0,168 to 1,427) | 0.5034 ^(1)^ |
| IL-12p40 | -1,095(-1,463 to0,005) | -1,303(-2,167 to -0,279) | 0.0107 |  | 2,723(2,293 to 2,972) | 2,578(0,477 to 3,237) | 0.0531 ^(1)^ |
| IL-15 | -2,081(-2,886 to-0,943) | -2,523(-3,523 to -1,824) | 0.0121 ^(1)^ |  | 0,169(-0,032 to 0,404) | 0,045(-0,743 to 0,444) | 0.0515 |
| IL-16 | -1,324(-1,924 to-1,042) | -1,151(-1,728 to -0,311) | 0.0160 ^(1)^ |  | 2,814(2,340 to 3,151) | 2,912(2,109 to 3,855) | 0.1902 ^(1)^ |
| IL-17A | -1,499(-2,119 to-1,249) | -1,439(-2,886 to -0,874) | 0.4940 |  | 0,909(0,491 to 1,881) | 0,940(0,123 to 2,059) | 0.6916 |
| IL-2 | -1,975(-2,174 to-1,818) | -2,081(-2,658 to -1,252) | 0.1748 |  | -0,656(-2,161 to 0,089) | -0,666(-2,161 to 0,367) | 0.8061 |
| CRP | 0,535(0,083 to0,938) | 0,743(-0,244 to 1,753) | 0.0552 ^(1)^ |  | 5,939(2,790 to 7,740) | 5,376(2,489 to 7,745) | 0.1402 |
| SAA | 1,303(1,058 to1,489) | 1,390(0,967 to 2,102) | 0.0074 ^(1)^ |  | 6,065(4,646 to 8,464) | 5,879(4,329 to 8,113) | 0.4609 |
| sICAM-1 | 0,363(0,276 to0,619) | 0,729(0,176 to 1,478) | < 0.0001 ^(1)^ |  | 5,859(2,821 to 5,956) | 5,835(3,605 to 6,107) | 0.6295 |
| sVCAM-1 | 0,573(0,368 to0,770) | 0,768(0,170 to 1,501) | 0.0004 ^(1)^ |  | 5,911(3,657 to 6,208) | 5,939(3,676 to 6,268) | 0.4438 |
| PIGF |  | | |  | 1,048(-0,091 to 1,221) | 1,111(0,211 to 1,417) | 0.0202 |
| VEGF-D |  | | |  | 2,817(2,362 to 3,055) | 2,841(2,221 to 3,111) | 0.4998 |
| CCL26 (Eotaxin-3) |  | | |  | 2,147(1,638 to 2,548) | 2,479(1,178 to 3,709) | 0.0002 |
| CCL11 (Eotaxin) |  |  |  |  | 2,637(2,294 to 2,887) | 2,538(1,132 to 2,905) | 0.4261 |
| IL-4 | nd | | |  | -4,000(-4,000 to -0,245) | -2,222(-4,000 to 0,140) | 0.0072 |
| CCL5 (RANTES) |  | | |  | 4,427(1,230 to 5,662) | 4,657(1,230 to 5,524) | 0.1608 |
| TNF-β |  | | |  | -0,126(-0,416 to 0,204) | -0,154(-1,507 to 0,579) | 0.7144 |
| TNF-α |  |  |  |  | 0,905(0,526 to 1,289) | 0,925(0,121 to 1,293) | 0.7693 |
| bFGF |  | | |  | 0,991(-0,523 to 1,813) | 0,760(-0,222 to 1,605) | 0.0936 |
| IFN-γ |  |  |  |  | 1,155(0,246 to 1,991) | 1,098(-0,086 to 2,473) | 0.4998 |
| IL-6 |  |  |  |  | -0,015(-2,745 to 0,448) | -0,194(-2,745 to 0,798) | 0.2641 |
| IL-10 |  | | |  | 0,126(-0,466 to 0,997) | 0,095(-0,858 to 0,743) | 0.4261 ^(1)^ |
|  |  | | |  |  |  |  |

^1^two-tailed Welch *t*-test (otherwise two-tailed Mann-Whitney test)

nd → not determined in the *SC*

< DL → more than 50 % of values bellow fit curve range

adjusted *p*-values → BH corrected *p*-values

| Angiogenesis markers | Th2 skewed markers | Markers of innate activation | Others |
| --- | --- | --- | --- |

**Table S3a.** Correlation (two-tailed Spearman's test) between cytokines/chemokines (log-transformed values) and oSCORAD/SCORAD in the *SC* and in plasma of children with AD.

| **logCYT** | **oSCORAD** | | | | | |  | **SCORAD** | | | | | |  |
| --- | --- | --- | --- | --- | --- | --- | --- | --- | --- | --- | --- | --- | --- | --- |
|  | ***SC* (*n* = 66)** | | | **Plasma (*n* = 47)** | | |  | ***SC* (*n* = 66)** | | | **Plasma (*n* = 47)** | | |  |
|  | ***r*** | **Adjusted *p*-value** | **95 % C.I.** | ***r*** | **Adjusted *p*-value** | **95 % C.I.** |  | **r** | **Adjusted *p*-value** | **95 % C.I.** | **r** | **Adjusted *p*-value** | **95 % C.I.** |  |
| Flt-1 | 0,4303 | 0,0019 | 0,2031 to 0,6135 | -0,0142 | 0,9413 | -0,3081 to 0,2822 |  | 0,4141 | 0,0028 | 0,1841 to 0,6011 | 0,0638 | 0,7692 | -0,2358 to 0,3524 |  |
| Tie-2 | -0,1530 | 0,2384 | -0,3810 to 0,0925 | -0,1779 | 0,3598 | -0,4495 to 0,1239 |  | 0,2553 | 0,0976 | 0,0068 to 0,4741 | -0,1841 | 0,3456 | -0,4546 to 0,1176 |  |
| VEGF-A | 0,4427 | 0,0014 | 0,2177 to 0,6230 | 0,0371 | 0,8719 | -0,2642 to 0,3318 |  | 0,4496 | 0,0012 | 0,2259 to 0,6282 | 0,0635 | 0,7692 | -0,2394 to 0,3551 |  |
| VEGF-C |  | < DL |  | -0,0349 | 0,8719 | -0,3267 to 0,2630 |  |  | < DL |  | -0,0545 | 0,8049 | -0,3442 to 0,2446 |  |
| CCL2 (MCP-1) | 0,4076 | 0,0034 | 0,1767 to 0,5960 | 0,0312 | 0,8897 | -0,2697 to 0,3265 |  | 0,3983 | 0,0044 | 0,1658 to 0,5889 | 0,0911 | 0,6709 | -0,2131 to 0,3792 |  |
| CCL22 (MDC) | 0,3625 | 0,0107 | 0,1249 to 0,5608 | 0,4888 | 0,0028 | 0,2229 to 0,6870 |  | 0,3730 | 0,0086 | 0,1368 to 0,5691 | 0,3968 | 0,0202 | 0,1116 to 0,6216 |  |
| CCL17 (TARC) | 0,4356 | 0,0017 | 0,2093 to 0,6176 | 0,3596 | 0,0425 | 0,0685 to 0,5943 |  | 0,4136 | 0,0028 | 0,1836 to 0,6007 | 0,3204 | 0,0788 | 0,0242 to 0,5648 |  |
| IL-5 | -0,2169 | 0,3181 | -0,4361 to 0,0266 | 0,4101 | 0,0160 | 0,1272 to 0,6313 |  | -0,1104 | 0,5063 | -0,3498 to 0,1424 | 0,4107 | 0,0160 | 0,1280 to 0,6317 |  |
| IL-13 | 0,2300 | 0,1474 | -0,0221 to 0,4546 | 0,2038 | 0,2934 | -0,1008 to 0,4734 |  | 0,1901 | 0,2424 | -0,0638 to 0,4209 | 0,2242 | 0,2474 | -0,0796 to 0,4898 |  |
| IL-1α | -0,2685 | 0,0785 | -0,4850 to -0,0209 |  | < DL |  |  | -0,1892 | 0,2424 | -0,4185 to 0,0627 |  | < DL |  |  |
| IL-18 | 0,4221 | 0,0022 | 0,1936 to 0,6072 | 0,2985 | 0,1072 | 4,895e-005 to 0,5481 |  | 0,3704 | 0,0090 | 0,1338 to 0,5671 | 0,3028 | 0,1015 | 0,0048 to 0,5514 |  |
| IL-1β | 0,1307 | 0,4376 | -0,1242 to 0,3694 | 0,2134 | 0,2718 | -0,0908 to 0,4812 |  | 0,2008 | 0,2183 | -0,0527 to 0,4300 | 0,2087 | 0,2806 | -0,0957 to 0,4774 |  |
| CXCL8 (IL-8) | 0,5338 | < 0,0001 | 0,3267 to 0,6920 | 0,4112 | 0,0160 | 0,1287 to 0,6321 |  | 0,4609 | 0,0009 | 0,2375 to 0,6380 | 0,4118 | 0,0487 | 0,1379 to 0,6272 |  |
| CXCL10 (IP-10) | 0,1094 | 0,5096 | -0,1434 to 0,3489 | -0,0206 | 0,9261 | -0,3171 to 0,2796 |  | 0,0868 | 0,6214 | -0,1657 to 0,3286 | -0,0169 | 0,9332 | -0,3138 to 0,2830 |  |
| CCL13 (MCP-4) | 0,0522 | 0,7693 | -0,1992 to 0,2972 | 0,4045 | 0,0177 | 0,1206 to 0,6272 |  | 0,0553 | 0,7633 | -0,1963 to 0,3001 | 0,3649 | 0,0385 | 0,0746 to 0,5982 |  |
| CCL3 (MIP-1α) | -0,1796 | 0,2641 | -0,4102 to 0,0726 | 0,4027 | 0,0183 | 0,1185 to 0,6259 |  | -0,1707 | 0,2887 | -0,4026 to 0,08168 | 0,3211 | 0,0785 | 0,0251 to 0,5654 |  |
| CCL4 (MIP-1β) | -0,0335 | 0,8593 | -0,2800 to 0,2172 | 0,1605 | 0,4261 | -0,1448 to 0,4379 |  | -0,0138 | 0,9332 | -0,2618 to 0,2360 | 0,1467 | 0,4665 | -0,1586 to 0,4264 |  |
| GM-CSF | 0,0193 | 0,9218 | -0,2307 to 0,2669 | 0,1387 | 0,4951 | -0,1667 to 0,4197 |  | 0,0800 | 0,6503 | -0,1724 to 0,3225 | 0,1174 | 0,5666 | -0,1876 to 0,4018 |  |
| IL-7 | -0,0721 | 0,6834 | -0,3154 to 0,1801 | 0,2084 | 0,2806 | -0,0960 to 0,4771 |  | -0,0018 | 0,9888 | -0,2506 to 0,2473 | 0,1911 | 0,3298 | -0,1137 to 0,4631 |  |
| IL-12p40 | 0,1395 | 0,3997 | -0,1134 to 0,3754 | 0,2013 | 0,4694 | -0,0981 to 0,4673 |  | 0,1827 | 0,2601 | -0,0694 to 0,4129 | 0,1759 | 0,5329 | -0,1241 to 0,4465 |  |
| IL-15 | -0,1511 | 0,3581 | -0,3855 to 0,1016 | 0,1042 | 0,6215 | -0,2005 to 0,3905 |  | -0,0994 | 0,5567 | -0,3399 to 0,1534 | 0,1349 | 0,5033 | -0,1704 to 0,4166 |  |
| IL-16 | 0,3079 | 0,0373 | 0,0639 to 0,5172 | 0,2722 | 0,1494 | -0,0285 to 0,5277 |  | 0,3189 | 0,0285 | 0,0760 to 0,5261 | 0,1779 | 0,3648 | -0,1272 to 0,4523 |  |
| IL-17A | 0,1642 | 0,3100 | -0,0883 to 0,3970 | 0,3021 | 0,0979 | 0,0077 to 0,5484 |  | 0,1910 | 0,2394 | -0,0609 to 0,4199 | 0,2972 | 0,1046 | 0,0023 to 0,5446 |  |
| IL-2 | 0,1029 | 0,5379 | -0,1499 to 0,3431 | 0,0498 | 0,8178 | -0,2524 to 0,3431 |  | 0,0736 | 0,6800 | -0,1786 to 0,3167 | 0,0731 | 0,7321 | -0,2304 to 0,3636 |  |
| CRP | 0,2019 | 0,2161 | -0,0516 to 0,4308 | 0,1580 | 0,4261 | -0,1439 to 0,4331 |  | 0,1697 | 0,2952 | -0,0848 to 0,4034 | 0,1688 | 0,3906 | -0,1330 to 0,4420 |  |
| SAA | 0,4517 | 0,0012 | 0,2266 to 0,6310 | 0,2148 | 0,2641 | -0,0858 to 0,4796 |  | 0,4328 | 0,0019 | 0,2041 to 0,6167 | 0,2105 | 0,2720 | -0,0904 to 0,4761 |  |
| sICAM-1 | 0,4781 | 0,0005 | 0,2583 to 0,6509 | -0,0650 | 0,7692 | -0,3565 to 0,2380 |  | 0,4623 | 0,0009 | 0,2392 to 0,6391 | -0,1135 | 0,5805 | -0,3984 to 0,1914 |  |
| sVCAM-1 | 0,4391 | 0,0017 | 0,2116 to 0,6215 | 0,3724 | 0,0337 | 0,0832 to 0,6037 |  | 0,4420 | 0,0016 | 0,2150 to 0,6237 | 0,3107 | 0,0927 | 0,0136 to 0,5575 |  |
| PIGF |  | | | -0,1248 | 0,5376 | -0,4080 to 0,1804 |  |  | | | -0,0940 | 0,6582 | -0,3817 to 0,2103 | |
| VEGF-D |  | | | 0,0352 | 0,8719 | -0,2627 to 0,3270 |  |  | | | 0,0211 | 0,9261 | -0,2759 to 0,3143 | |
| CCL26 (Eotaxin-3) |  | | | 0,3314 | 0,0646 | 0,0401 to 0,5708 |  |  | | | 0,4220 | 0,0119 | 0,1448 to 0,6378 | |
| CCL11 (Eotaxin) |  |  |  | 0,1561 | 0,4376 | -0,1493 to 0,4343 |  |  |  |  | 0,1131 | 0,5805 | -0,1918 to 0,3981 | |
| IL-4 | nd | | | -0,0756 | 0,7231 | -0,3627 to 0,2247 |  | nd | | | -0,0350 | 0,8719 | -0,3268 to 0,2630 | |
| CCL5 (RANTES) |  | | | -0,0169 | 0,9332 | -0,3106 to 0,2797 |  |  | | | -0,0207 | 0,9269 | -0,3140 to 0,2762 | |
| TNF-β |  | | | -0,0027 | 0,9505 | -0,3009 to 0,2960 |  |  | | | -0,0150 | 0,9088 | -0,3120 to 0,2847 | |
| TNF-α |  | | | 0,5008 | 0,0020 | 0,2413 to 0,6935 |  |  | | | 0,4039 | 0,0165 | 0,1234 to 0,6247 | |
| bFGF |  | | | -0,0829 | 0,6916 | -0,3691 to 0,2176 |  |  | | | -0,1121 | 0,5805 | -0,3942 to 0,1893 | |
| IFN-γ |  |  |  | 0,2679 | 0,1507 | -0,0297 to 0,5219 |  |  |  |  | 0,2844 | 0,1252 | -0,0118 to 0,5348 | |
| IL-6 |  |  |  | 0,2799 | 0,1325 | -0,0167 to 0,5312 |  |  |  |  | 0,2904 | 0,1152 | -0,0052 to 0,5394 | |
| IL-10 |  |  |  | 0,2636 | 0,1578 | 0,0342 to 0,5185 |  |  |  |  | 0,1930 | 0,3180 | -0,1083 to 0,4619 | |

nd → not determined in the *SC*

< DL → more than 50 % of values bellow fit curve range

adjusted *p*-values → BH corrected *p*-values

| Angiogenesis markers | Th2 skewed markers | Markers of innate activation | Others |
| --- | --- | --- | --- |

**Table S3b.** Correlation (two-tailed Spearman's test) between cytokines/chemokines (log-transformed values)

and TEWL in the *SC* and in plasma of children with AD.

| **logCYT** | **TEWL** | | | | | |
| --- | --- | --- | --- | --- | --- | --- |
|  | ***SC* (*n* = 66)** | | | **Plasma (*n* = 47)** | | |
|  | ***r*** | **Adjusted *p*-value** | **95 % C.I.** | ***r*** | **Adjusted *p*-value** | **95 % C.I.** |
| Flt-1 | 0,6716 | < 0,0001 | 0,5060 to 0,7895 | -0,1539 | 0,4438 | -0,4325 to 0,1515 |
| Tie-2 | 0,2270 | 0,1507 | -0,0253 to 0,4521 | -0,2276 | 0,2424 | -0,4926 to 0,0760 |
| VEGF-A | 0,5624 | < 0,0001 | 0,3627 to 0,7127 | -0,0980 | 0,6503 | -0,3883 to 0,2099 |
| VEGF-C |  | < DL |  | 0,0754 | 0,7254 | -0,2281 to 0,3656 |
| CCL2 (MCP-1) | 0,2598 | 0,0936 | 0,0096 to 0,4794 | 0,1927 | 0,3302 | -0,1158 to 0,4673 |
| CCL22 (MDC) | 0,4374 | 0,0017 | 0,2095 to 0,6202 | 0,5639 | 0,0005 | 0,3159 to 0,7398 |
| CCL17 (TARC) | 0,4910 | 0,0004 | 0,2739 to 0,6605 | 0,5981 | 0,0002 | 0,3616 to 0,7623 |
| IL-5 | -0,2462 | 0,1152 | -0,4682 to 0,0049 | 0,2590 | 0,1789 | -0,0464 to 0,5201 |
| IL-13 | 0,0116 | 0,9414 | -0,2419 to 0,2637 | 0,2337 | 0,2384 | -0,0732 to 0,5002 |
| IL-1α | -0,3735 | 0,0090 | -0,5709 to -0,1353 |  | < DL |  |
| IL-18 | 0,7340 | < 0,0001 | 0,5922 to 0,8317 | 0,3278 | 0,0765 | 0,0289 to 0,5729 |
| IL-1β | 0,1142 | 0,5022 | -0,1427 to 0,3568 | 0,1502 | 0,4609 | -0,1588 to 0,4323 |
| CXCL8 (IL-8) | 0,5763 | < 0,0001 | 0,3786 to 0,7237 | 0,2139 | 0,2751 | -0,0939 to 0,4844 |
| CXCL10 (IP-10) | 0,2403 | 0,1269 | -0,0113 to 0,4632 | 0,0049 | 0,9779 | -0,2973 to 0,3062 |
| CCL13 (MCP-4) | 0,0449 | 0,8061 | -0,2084 to 0,2924 | 0,5216 | 0,0016 | 0,2609 to 0,7114 |
| CCL3 (MIP-1α) | -0,2165 | 0,1748 | -0,4433 to 0,03631 | 0,2342 | 0,2384 | -0,0726 to 0,5006 |
| CCL4 (MIP-1β) | -0,2729 | 0,0765 | -0,4902 to -0,0237 | 0,2214 | 0,2616 | -0,0861 to 0,4904 |
| GM-CSF | -0,1899 | 0,2424 | -0,4207 to 0,0640 | 0,4192 | 0,0152 | 0,1345 to 0,6400 |
| IL-7 | -0,1529 | 0,3573 | -0,3888 to 0,1019 | 0,1659 | 0,4157 | -0,1430 to 0,4454 |
| IL-12p40 | 0,0052 | 0,9741 | -0,2459 to 0,2558 | 0,2424 | 0,3876 | -0,0677 to 0,5099 |
| IL-15 | -0,1885 | 0,2463 | -0,4195 to 0,0655 | 0,0875 | 0,6837 | -0,2201 to 0,3792 |
| IL-16 | 0,2954 | 0,0498 | 0,0481 to 0,5086 | 0,5299 | 0,0013 | 0,2716 to 0,7170 |
| IL-17A | 0,1688 | 0,2974 | -0,0857 to 0,4026 | 0,4753 | 0,0041 | 0,2061 to 0,6776 |
| IL-2 | 0,0379 | 0,8350 | -0,2150 to 0,2861 | -0,0756 | 0,7254 | -0,3690 to 0,2314 |
| CRP | 0,1941 | 0,2394 | -0,0617 to 0,4261 | 0,1036 | 0,6220 | -0,2011 to 0,3899 |
| SAA | 0,2813 | 0,0678 | 0,0307 to 0,4987 | 0,0481 | 0,8238 | -0,2540 to 0,3416 |
| sICAM-1 | 0,4206 | 0,0028 | 0,1878 to 0,6087 | -0,1469 | 0,4694 | -0,4296 to 0,1620 |
| sVCAM-1 | 0,3905 | 0,0064 | 0,1528 to 0,5856 | 0,2570 | 0,1827 | -0,0485 to 0,5185 |
| PIGF |  | | | -0,0892 | 0,6810 | -0,3807 to 0,2185 |
| VEGF-D |  | | | 0,1264 | 0,5329 | -0,1787 to 0,4094 |
| CCL26 (Eotaxin-3) |  | | | 0,4103 | 0,0160 | 0,1275 to 0,6315 |
| CCL11 (Eotaxin) |  |  |  | 0,5146 | 0,0018 | 0,2520 to 0,7066 |
| IL-4 | nd | | | 0,0511 | 0,8141 | -0,2512 to 0,3443 |
| CCL5 (RANTES) |  | | | -0,0527 | 0,8087 | -0,3457 to 0,2497 |
| TNF-β |  | | | 0,3303 | 0,1494 | 0,0317 to 0,5748 |
| TNF-α |  | | | 0,1796 | 0,3598 | -0,1256 to 0,4537 |
| bFGF |  | | | -0,0956 | 0,6528 | -0,3831 to 0,2088 |
| IFN-γ |  | | | 0,0552 | 0,8049 | -0,2473 to 0,3479 |
| IL-6 |  |  |  | 0,0350 | 0,8719 | -0,2662 to 0,3300 |
| IL-10 |  |  |  | 0,2390 | 0,2185 | -0,0640 to 0,5017 |

nd → not determined in the *SC*

< DL → more than 50 % of values bellow fit curve range

adjusted *p*-values → BH corrected *p*-values

| Angiogenesis markers | Th2 skewed markers | Markers of innate activation | Others |
| --- | --- | --- | --- |
