# Supplementary material for: Systemic and stratum corneum biomarkers of severity in infant atopic dermatitis include markers of innate and T helper cell‐related immunity and angiogenesis
Source: Br J Dermatol. 2018 Oct 4;180(3):586–96. doi: 10.1111/bjd.17088 (PMC6446820; doi:10.1111/bjd.17088)
Supplement: Supplementary file 4 — Fig S3. Levels of cytokines and chemokines, natural moisturizing factor and transepidermal water loss in healthy controls and children with atopic dermatitis stratified for FLG mutations. [file BJD-180-586-s004.tif]

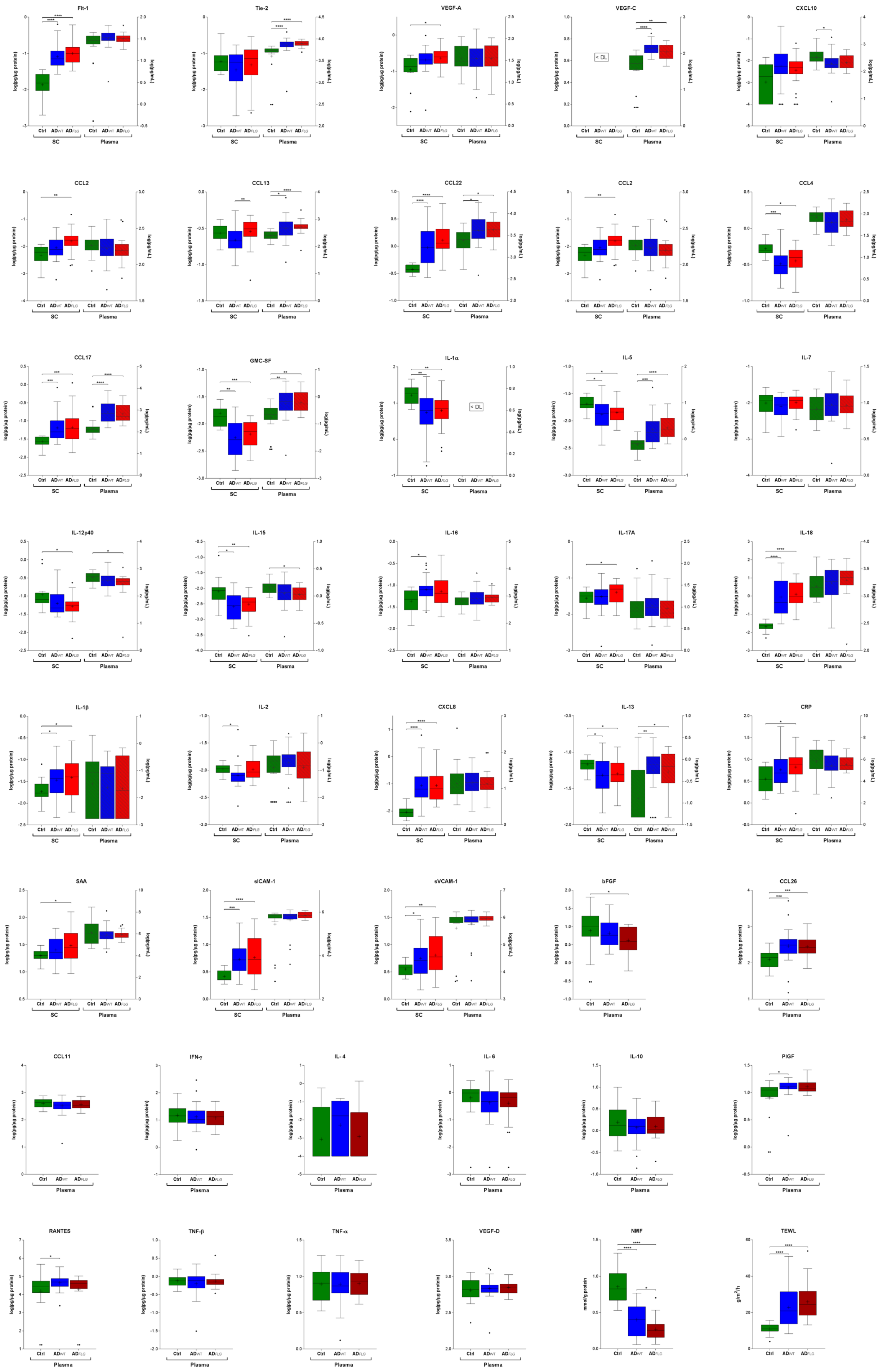

Cytokines/chemokines SC: $n(AD_{WT}) = 30$, $n(AD_{FLG}) = 32$, $n(Ctrl) = 13$; plasma: $n(AD_{WT}) = 25$, $n(AD_{FLG}) = 21$, $n(Ctrl) = 20$
NMF: SC: $n(AD_{WT}) = 37$, $n(AD_{FLG}) = 32$, $n(Ctrl) = 18$; TEWL: $n(AD_{WT}) = 35$, $n(AD_{FLG}) = 32$, $n(Ctrl) = 19$
